# Potential Natural Inhibitors of MRSA ABC Transporters and MecA Identified Through In Silico Approaches

**DOI:** 10.3390/microorganisms13061431

**Published:** 2025-06-19

**Authors:** Benson Otarigho, Paul M. Duffin, Mofolusho O. Falade

**Affiliations:** 1 Department of Genetics, The University of Texas, MD Anderson Cancer Center, Houston, TX 77030, USA; pduffin@transy.edu; 2 Department of Biology, Transylvania University, Lexington, KY 40508, USA

**Keywords:** Methicillin-resistant *Staphylococcus aureus* (MRSA), multidrug ABC transporter, MecA, antibiotic resistance, molecular docking, natural compounds

## Abstract

Methicillin-resistant *Staphylococcus aureus* (MRSA) poses a significant clinical challenge due to its resistance to multiple antibiotics. The urgent need for new therapeutic approaches has led to the exploration of natural compounds as potential treatments, particularly those targeting the key bacterial proteins involved in antibiotic resistance. This study focused on the multidrug ABC transporter and MecA proteins, which play crucial roles in MRSA′s pathogenicity and resistance mechanisms. Using computational techniques and molecular docking methods, we assessed the interactions of 80 natural compounds with *S. aureus* multidrug ABC transporter SAV1866 (SAV1866) and MecA proteins. Our analysis revealed 14 compounds with robust binding to SAV1866 and one compound with a strong affinity for MecA. Notably, these compounds showed weaker affinities for the MgrA, MepR, and arlR proteins, suggesting specificity in their interactions. Among the 15 promising compounds identified, 1′,2-Binaphthalen-4-one-2′,3-dimethyl-1,8′-epoxy-1,4′,5,5′,8,8′-hexahydroxy-8-O-β-glucopyranosyl-5′-O-β-xylopyranosyl(1→6)-β-glucopyranoside; Cis-3,4-dihydrohamacanthin b; and Mamegakinone exhibited the highest binding affinities to *S. aureus* SAV1866. These compounds represent diverse chemical classes, including alkaloids, indole derivatives, naphthalenes, and naphthoquinones, offering a range of structural scaffolds for further drug development. Our findings provide valuable insights into potential new antibacterial agents targeting *S. aureus* SAV1866 and MecA proteins. These results lay the groundwork for future in vitro and in vivo studies to validate these compounds′ efficacy for combating MRSA infections, potentially leading to the development of novel therapeutic strategies against antibiotic-resistant bacteria.

## 1. Introduction

Methicillin-resistant *Staphylococcus aureus* (MRSA) is a major cause of nosocomial and community-acquired infections worldwide [1,2,3,4]. The overuse and misuse of antibiotics are among the factors that have contributed to the development of antibiotic resistance [5,6]. MRSA poses a significant public health challenge, as it is resistant to many antibiotics that are commonly used to treat bacterial infections [1,4]. This resistance is primarily due to the presence of an *mecA* gene, which encodes a protein called PBP2a that enables bacteria to evade the effects of many antibiotics [7,8,9,10]. Another critical protein involved in antibiotic resistance is the multidrug ABC transporter SAV1866. This protein is responsible for the active efflux of a broad spectrum of antibiotics from bacterial cells, utilizing energy derived from ATP hydrolysis. By actively pumping out antimicrobial agents, SAV1866 reduces the intracellular concentration of these drugs, thereby decreasing their efficacy and contributing significantly to the survival of bacteria in the presence of antibiotics [11,12,13,14,15]. Furthermore, the genome of *S. aureus* also contains other genes that encode antibiotic resistance proteins, such as MgrA, MepR, and arlR [13,16]. Although some antibiotics, such as vancomycin and linezolid, are relatively effective against MRSA [3], the treatment of infections can be difficult due to the emergence of new resistant strains [4,17]. Thus, there is an urgent need for alternative treatments and the discovery of new medications or therapeutic approaches to combat MRSA [18].

Natural agents, including plant extracts and essential oils, hold great promise as alternative therapies for treating bacterial infections [19,20]. These agents have an extensive history of traditional medicinal usage and have demonstrated substantial antibacterial potency [19,20,21]. A comprehensive body of research provides compelling evidence substantiating the efficacy of natural agents for treating bacterial pathogens [19]. Remarkably, these natural agents have displayed efficacy against both Gram-positive and Gram-negative bacterial strains, including antibiotic-resistant strains such as MRSA [3,19,22,23].

The unique effectiveness of natural agents against bacterial infections often emanates from their distinct mechanisms compared to those of antibiotics [6,22]. These mechanisms include disrupting bacterial cell membranes, suppressing enzyme activity, and interrupting bacterial DNA replication [22]. By simultaneously targeting multiple cellular sites, these compounds aid in the disruption of vital cellular processes, thereby inhibiting bacterial proliferation; diminishing their pathogenicity; and enhancing their susceptibility to host immune responses, an important arm of chemotherapeutic efficacy [6]. Furthermore, natural agents have exhibited efficacy against a spectrum of bacterial infections, encompassing pneumonia, urinary tract infections, and cutaneous infections [22]. Additionally, natural agents may yield fewer adverse effects when compared to antibiotics, largely stemming from their selective toxicity against bacterial cells while leaving human cells untouched [22,23].

Consequently, the investigation of natural compounds as multi-target therapeutics against bacterial pathogens provides a promising area for identifying new drugs. These compounds exhibit a diverse array of chemical structures and mechanisms of action, making them attractive sources for novel chemotherapeutics [22,23]. Given these advantages, further research is required to identify natural compounds possessing antibacterial properties, particularly those demonstrating a robust affinity to well-known MRSA antibiotic resistance-associated proteins, such as MecA, SAV1866, MgrA, MepR, and arlR [24,25].

In recent years, computational drug discovery has emerged as a vital preliminary strategy for identifying bioactive compounds [25,26]. Integrating in silico approaches, such as molecular docking and virtual screening, enables the rapid and cost-effective evaluation of chemical libraries against molecular targets of interest [10,25,27,28,29,30]. This is particularly relevant in the context of antibiotic resistance, where the discovery of novel inhibitors targeting resistance-associated proteins is urgent but resource-intensive [27,28,31]. Computational screening allows for the prioritization of compounds with favorable binding properties, thus narrowing the scope for subsequent in vitro and in vivo validation. By focusing on well-defined protein targets [16], virtual screening provides an efficient starting point to accelerate the identification of potential inhibitors that may overcome the drug resistance of *S. aureus*.

In this study, we aimed to explore the efficacy of natural compounds derived from the South African Natural Compounds Database (SANCDB) [32] against the major antibiotic resistance proteins found in MRSA. Some of these proteins, namely MecA, SAV1866, MgrA, MepR, and arlR, are known to contribute significantly to the development of antibiotic resistance in MRSA strains [16]. These proteins were included in our screening pipeline to evaluate whether natural compounds could simultaneously disrupt multiple, complementary resistance pathways. This dual-target strategy was intended to expand the scope of potential inhibitors, thereby increasing the likelihood of identifying compounds with enhanced efficacy against MRSA resistance. To assess the binding capabilities of these natural compounds, we utilized a molecular docking screening technique. Our analysis revealed that most of the natural compounds exhibited binding affinity towards the MRSA multidrug ABC transporter protein, which is a crucial component in the efflux of drugs from MRSA cells [11,15]. These compounds primarily belong to diverse chemical groups, such as binaphthalenones, glycosides, organooxygens, alkaloids, indoles, carboxylic acids, naphthoquinones, naphthalenes, and flavonoids [32,33,34,35,36,37,38,39]. Additionally, our study identified a specific compound that displayed a remarkable affinity for the MecA protein, a key regulator of MRSA virulence [8,9,39]. The strong binding demonstrated between this natural compound and MecA indicates its potential as an inhibitor of MecA-mediated virulence, which could potentially reduce the pathogenicity of MRSA strains. Our work provides valuable insights into using natural compounds as inhibitors of antibiotic resistance proteins in MRSA. By targeting these proteins, natural compounds hold promise as potential therapeutic agents to combat MRSA infections and overcome antibiotic resistance.

## 2. Materials and Methods

### 2.1. Data Mining and Retrieval of Antibiotic Resistance Proteins

This study focused on antibiotic resistance proteins, which play significant roles as global regulators of and major virulence determinants in *S. aureus* [7,8,9,13,14,15]. Specifically, the multidrug ABC transporter SAV1866, MecA, MgrA, MepR, and arlR were of interest, and their structural information was obtained from the Protein Data Bank (PDB), accessible at https://www.rcsb.org/ (accessed on 12 February 2025) [40,41]. The downloaded experimental data snapshots of the protein structures from the PDB provided specific details about the methods employed for their structural determination, along with the relevant metrics. The experimental data snapshot of SAV1866 (PDB ID: 2HYD) and MecA (PDB ID: 1MWS) indicated an X-ray diffraction method with a resolution of 3.00 Å and 2.00 Å, respectively. The R-Value Free, R-Value Work, and R-Value reported for SAV1866 were 0.272, 0.255, and 0.255, respectively [12], while the R-Value Free, R-Value Work, and R-Value reported for MecA were 0.274, 0.234, and 0.234, respectively [42]. Similarly, for MgrA (PDB ID: 2BV6), the experimental data snapshot indicated an X-ray diffraction method with a resolution of 2.80 Å, and the R-Value Free, R-Value Work, and R-Value Observed were reported as 0.292, 0.250, and 0.250, respectively [43]. For MepR (PDB ID: 4L9N), the experimental data snapshot indicated an X-ray diffraction method with a resolution of 1.60 Å, and the R-Value Free, R-Value Work, and R-Value Observed were reported as 0.217, 0.190, and 0.191, respectively [44]. For arIR (PDB ID: 6IS3), the experimental data snapshot indicated an X-ray diffraction method with a resolution of 1.55 Å, and the R-Value Free, R-Value Work, and R-Value Observed were reported as 0.206, 0.173, and 0.176, respectively [45]. These experimental data snapshots offer valuable insights into the quality and reliability of the obtained protein structures. The resolution values indicate the level of detail at which the structures were determined, while the R-values provide an assessment of the agreement between the experimental data and the refined protein models [46,47].

### 2.2. Data Mining and Retrieval of Natural Compounds with Known Antibiotic Activities

The exploration for antibacterial compounds was carried out using the SANCDB, which comprises 1012 natural compounds [32,48], and was accessed at https://sancdb.rubi.ru.ac.za/ (accessed on 1 February 2025). The compounds chosen for the search were already recognized for their antibiotic properties against a diverse range of bacteria (Diallo, et al., 2021) [32]. The SANCDB serves as a comprehensive and freely accessible repository of natural chemical compounds originating from South African biodiversity [32,48]. Eighty compounds were selected and mined in the PDB format. Their 2D chemical structures were analyzed and obtained using Marvin JS version 23.15.5 (https://marvinjs-demo.chemaxon.com/latest/index.html, accessed on 20 February 2025), an online chemical editor developed by ChemAxon that enables users to draw and visualize chemical structures on the web.

### 2.3. Docking of the Natural Compounds Against the MRSA Antibiotic Resistance Protein

Each of the 80 compounds selected from the 1012 natural compounds available in the SANCDB was subjected to molecular docking against SAV1866, MecA, MgrA, MepR, and arlR using the CB-Dock2 server, accessible at https://cadd.labshare.cn/cb-dock2/php/blinddock.php (accessed on 12 February 2025) [49]. Prior to molecular docking, potential binding cavities on the distinct MRSA antibiotic resistance proteins were predicted using the CB-Dock2 server, which automatically identified the most suitable binding sites for each compound. Only compounds that exhibited a strong affinity, with vina scores of −10 kcal/mol and below, were selected for further analysis. To validate the binding affinities of the selected compounds, additional docking was performed using the DockThor tool, at https://dockthor.lncc.br/v2/ (accessed on 1 June 2025) [50]. The selection of compounds with a high binding affinity was crucial, as it indicated a stronger potential for specific interactions with the target proteins. These compounds were more likely to exhibit favorable binding and potentially possess greater therapeutic efficacy against the target proteins.

### 2.4. Analyses of the Binding Affinity of the Antibiotic Resistance Proteins to the Natural Compounds

In this study, we utilized computational tools to analyze and visualize the binding affinities of 80 natural compounds against SAV1866, MecA, MgrA, MepR, and arlR. The vina scores were visualized using the Datawrapper tool (https://www.datawrapper.de/, accessed on 1 March 2025). By inputting the vina scores into Datawrapper, we generated a chart to represent the binding affinities of the 80 compounds against the target proteins. This visualization provided an overview of the compound–protein interactions and allowed for the easy comparison and interpretation of the data. The significant binding affinities, with a vina score of less than −10 kcal/mol, were further analyzed with Datawrapper. This visualization provided a more detailed and focused representation of the interactions between the identified compounds and proteins. Additionally, we employed TBtool [51], which was downloaded from https://github.com/CJ-Chen/TBtools, (accessed on 1 March 2025), to create a heatmap to visualize the 15 natural compounds that were significantly bound to SAV1866 and MecA. The affinities of these 15 compounds to the MgrA and MepR proteins were also included in this analysis. The heatmap representation allowed us to observe the patterns and trends in the binding affinities of these compounds across multiple proteins simultaneously. This visualization provided a comprehensive view of the compound–protein interactions and highlighted the compounds that exhibited high binding affinity to multiple target proteins.

## 3. Results

**Figure 1:**
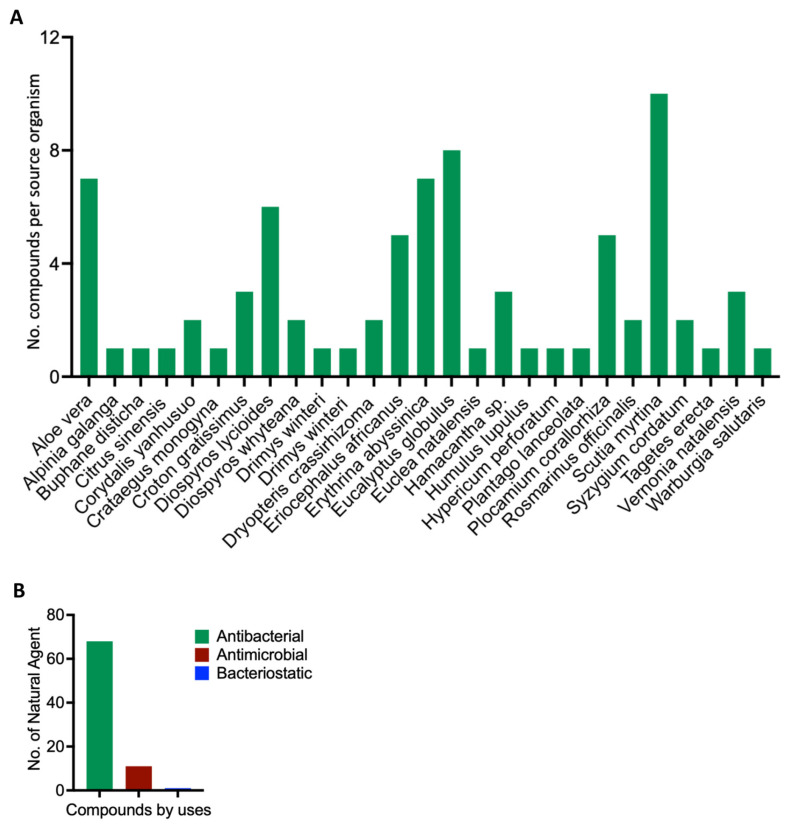
Scientific depiction of the biological and botanical characteristics of the natural compounds found within the South African Natural Compounds Database. (**A**) The plant sources that yield these compounds, highlighting the diverse botanical sources present in the South African Natural Compounds Database. (**B**) The different types of activity exhibited by the compounds.

After mining the SANCDB, we identified 80 compounds sourced from tropical plants (Supplementary Tables S1–S5). These compounds are sourced from different plant species, including *Scutia myrtina*, *Erythrina abyssinica*, *Eucalyptus globulus*, and *Aloe vera,* which have the highest number of compounds with antibacterial activities (Figure 1A). Among these naturally derived compounds, 68 have confirmed antibacterial properties, while 11 and 1, respectively, exhibit antimicrobial and bacteriostatic properties (Figure 1B and Supplementary Tables S1–S5). This suggests that the activities described for these plants make them a rich repository of bioactive compounds with antibacterial potential.

**Figure 2:**
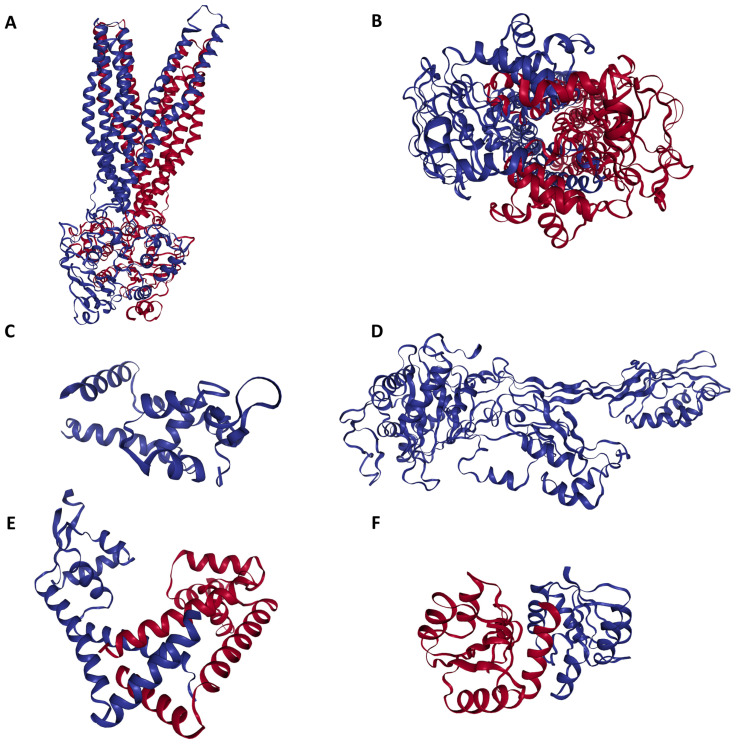
Three-dimensional configuration of proteins responsible for conferring antibiotic resistance to methicillin-resistant Staphylococcus aureus (MRSA). (**A**) Side view of MRSA SAV1866: This view showcases the MRSA SAV1866 protein from a lateral perspective, highlighting its structural chains of transmembrane proteins. (**B**) Dorsal view of MRSA SAV1866: This view presents the MRSA SAV1866 protein from a top-down or dorsal perspective, showing the aperture of the transmembrane proteins. (**C**) Structure of MRSA MgrA, displaying the detailed structure of the MRSA MgrA protein. (**D**) Structure of MRSA MecA. (**E**) Structure of MRSA MepR. (**F**) Structure of MRSA arIR.

**Figure 3:**
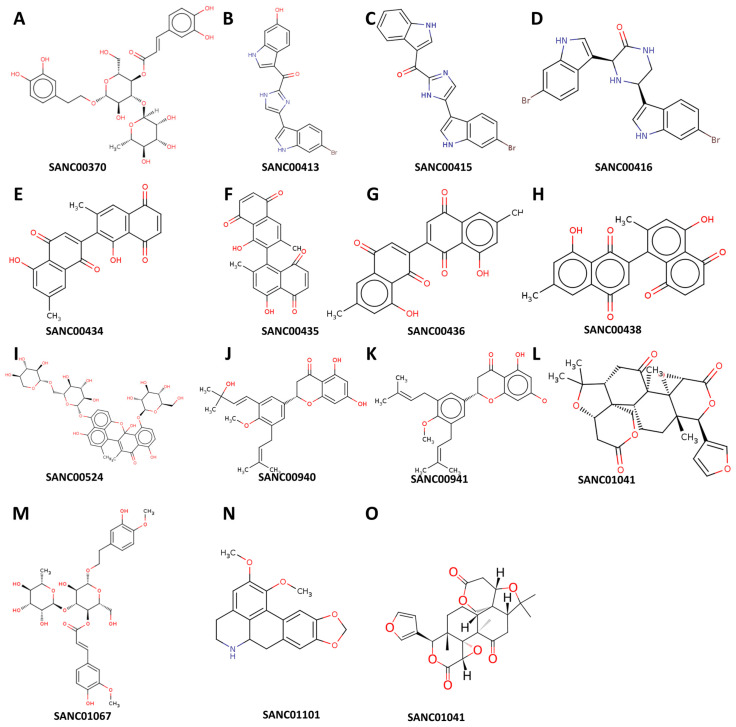
Structural representation of the natural compounds obtained from the South African Natural Compounds Database that exhibit strong binding affinity to two specific proteins, namely MRSA SAV1866 and MecA, which are associated with methicillin-resistant *Staphylococcus aureus* (MRSA) resistance.

**Figure 4:**
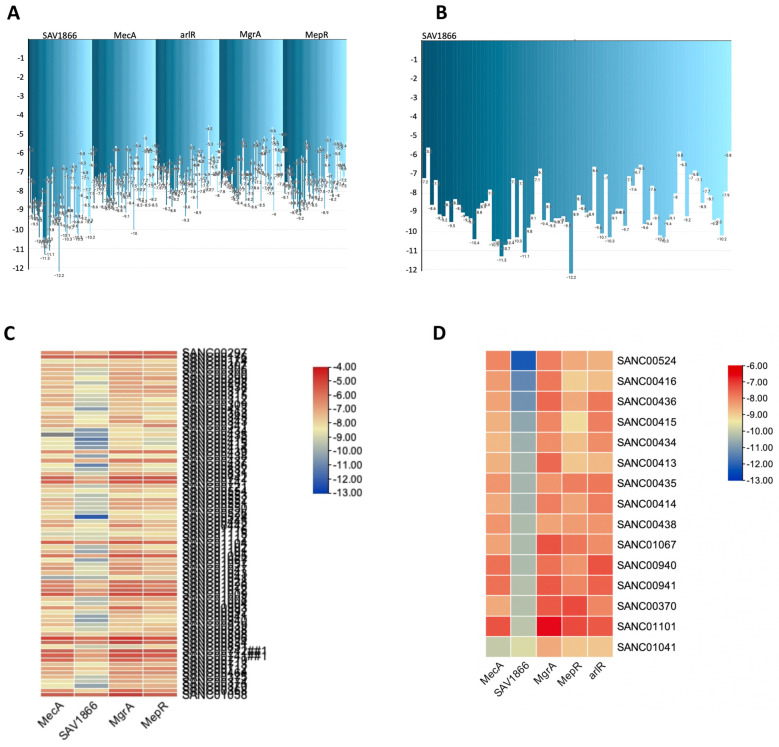
A comprehensive view of the natural compounds that exhibited robust binding affinity to the MRSA SAV1866, MecA, arlR, MgrA, and MepR proteins. (**A**) Inverted bar chart illustrating the bonding of 80 compounds to MRSA SAV1866, MecA, arlR, MgrA, and MepR. (**B**) Inverted bar chart depicting the bonding of 14 compounds to MRSA SAV1866, since only the SAV1866 proteins demonstrated strong bonding with more than one compound. (**C**) Heatmap visualizing the bonding of 80 compounds to MRSA SAV1866, MecA, arlR, MgrA, and MepR. The heatmap employs a color gradient to indicate the strength of the bonding interaction, enabling the easy identification of compounds with a high or low affinity for each protein. (**D**) Heatmap illustrating the bonding of 15 compounds to MRSA SAV1866, MecA, arlR, MgrA, and MepR. This component specifically focuses on the heatmap representation of the binding affinity of 15 compounds with the MRSA SAV1866, MecA, arlR, MgrA, and MepR proteins. The color-coded heatmap visually represents the compounds′ bonding strengths with MRSA SAV1866, MecA, arlR, MgrA, and MepR.

**Figure 5:**
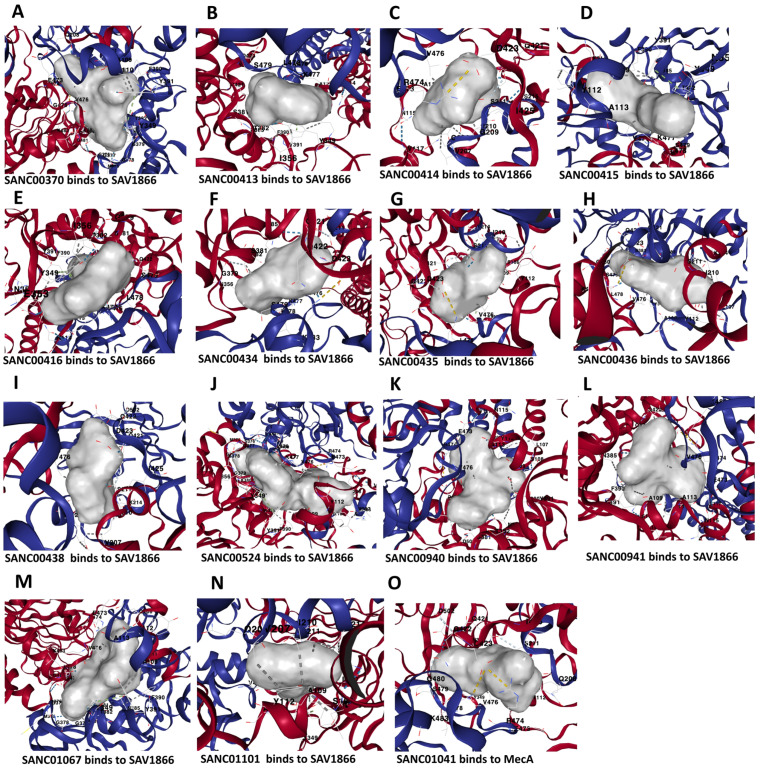
Three-dimensional binding structure of 14 natural compounds to SAV1866 and that of the compound SANC01041 (Limonin) to the MecA proteins of methicillin-resistant *Staphylococcus aureus* (MRSA).

**Figure 6:**
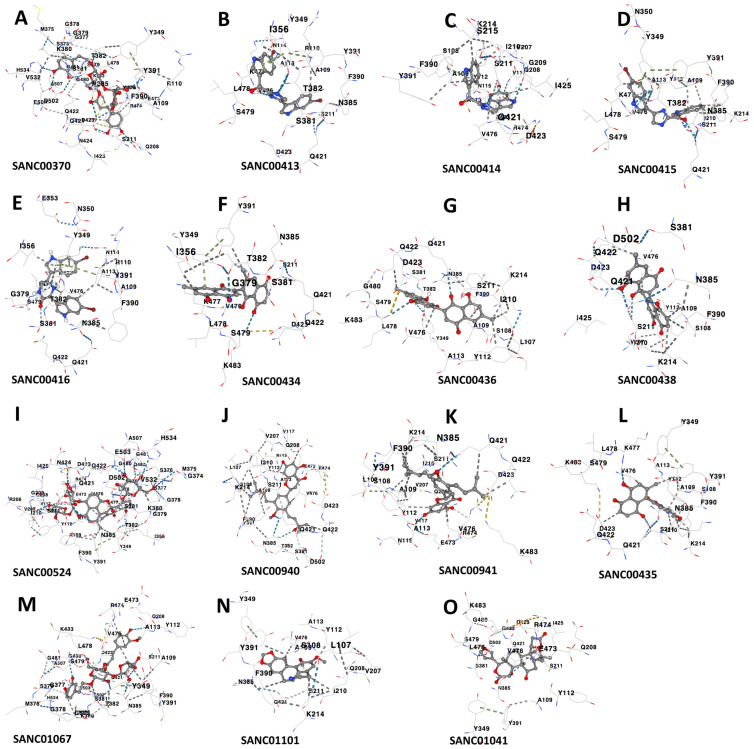
Interacting residues between the multidrug ATP-binding cassette (ABC) transporter (SAV1866) and each of the 14 docked natural compounds, as well as the interactions between MecA and its binding compound. Panels (**A**–**N**) show the key amino acid residues at the binding interface of SAV1866 with each compound, while Panel (**O**) displays the interacting residues for MecA.

We used molecular docking to evaluate the interactions of the 80 compounds with the five protein structures, SAV1866, MecA, MgrA, MepR, and arlR, which were obtained from the PDB database (Figure 2). We calculated the binding affinities, with vina scores less than −10 kcal/mol used for selecting the active compounds (Supplementary Tables S1–S5). The 2D structures of the 15 compounds that demonstrated significant binding affinity are shown in Figure 3. Among these, 14 compounds displayed robust interactions with SAV1866, with only one compound exhibiting a substantial interaction with MecA (Figure 3, Figure 4, Figure 5 and Figure 6, Table 1, and Supplementary Tables S1–S7). The specific residues involved in the interactions of the compounds with SAV1866 and MecA are depicted in Figure 6.

It should be noted that the docking affinity scores generated by different computational tools, such as CB-Dock2 (used as the primary tool) and DockThor (used for validation), cannot be directly compared in absolute terms. For instance, while CB-Dock2 predicted the binding affinity of licofelone to the CB2 receptor as −8.1 kcal/mol [52], DockThor calculated a lower affinity of −6.452 kcal/mol for the same protein–ligand pair, a pattern also observed in the current study′s validation results (Supplementary Table S8). This discrepancy occurs because each docking program uses distinct scoring functions, search algorithms, and parameter calibrations to estimate the binding energies [31,50,53]. However, following validation, all the selected compounds were confirmed as promising candidates.

**Figure 7:**
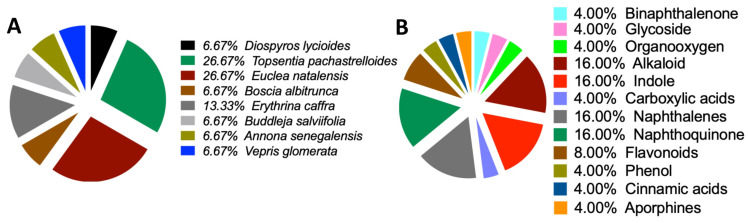
Origin sources of the compounds that demonstrated strong binding to the MRSA antibiotics resistance protein. (**A**) Sources of the compounds that strongly bind to MRSA multidrug ATP-binding cassette (ABC) and MecA. (**B**) Classification of the compounds that strongly bind to MRSA multidrug ATP-binding cassette (ABC) and MecA.

Consequently, none of the 80 compounds docked showed a strong affinity for MgrA, MepR, or ArlR, and thus were not further investigated because of their high vina scores, which were greater than −10 kcal/mol. To provide further insights into the activities of the docked compounds that had the strongest affinity, we decided to explore their plant sources. Our work revealed that the following plant species—*Diospyros lycioides*, *Topsentia pachastrelloides*, *Euclea natalensis*, *Boscia albitrunca*, *Erythrina caffra*, *Buddleja salviifolia*, *Annona senegalensis*, and *Vepris glomerata*—were the sources of the identified compounds (Figure 7A and Supplementary Table S7). Each of these plant species is known to produce compounds that exhibit antibacterial properties [33,34,35,36,37,38,39]. Among the identified plant species, *Topsentia pachastrelloides* and *Euclea natalensis* stood out, as they had the highest number of compounds with antibacterial activities, with each species contributing four compounds (Figure 7A). The compounds with the highest binding affinities were SANC00524 (1′,2-Binaphthalen-4-one-2′,3-dimethyl-1,8′-epoxy-1,4′,5,5′,8,8′-hexahydroxy-8-O-β-glucopyranosyl-5′-O-β-xylopyranosyl(1→6)-β-glucopyranoside), SANC00416 (Cis-3,4-dihydrohamacanthin b), and SANC00436 (Mamegakinone). Their vina scores were − 12.2 kcal/mol, -11.3 kcal/mol, and − 11.1 kcal/mol, respectively (Figure 4, Table 1, and Supplementary Tables S6 and S7). These strong binding affinities suggested their therapeutic potential. Further analyses showed that most of the compounds identified belonged to various chemical classes, such as alkaloids, indole derivatives, naphthalenes, and naphthoquinones (Figure 7B and Supplementary Table S7). Previous work has demonstrated that these diverse chemical classes exhibit antibacterial properties against various bacteria and some fungi [24,54,55,56].

## 4. Discussion

The current study employed molecular docking techniques to investigate the interaction between natural agents and different bacterial proteins, namely SAV1866, MecA, MgrA, MepR, and arlR, each of which is associated with well-established but distinct mechanisms of antibiotic resistance, survival, and pathogenicity in MRSA. By targeting multiple proteins, a multi-target screening approach was adopted to improve the likelihood of identifying compounds with broad-spectrum or synergistic antibacterial effects. The availability of high-resolution 3D protein structures contributed to the accuracy of the docking results and allowed for a meaningful analysis of the ligand–protein interactions. Here, we screened a total of 80 natural compounds against major MRSA antibiotic resistance proteins. The results revealed that most of these compounds exhibited high affinity towards the MRSA multidrug ABC transporter and one compound demonstrated a strong affinity against MecA. Our current work emphasizes the potential of natural agents as antibacterial therapies and provides valuable insights into their mechanisms of action.

SAV1866 is a multidrug ABC transporter protein that plays a crucial role in expelling antibiotics and toxic substances from bacterial cells. This efflux activity of SAV1866 contributes to multidrug resistance and reduces the effectiveness of antimicrobial treatments [11,12,13,15]. Compounds that bind strongly to SAV1866 have the potential to inhibit its function and counteract its antibiotic efflux [57]. By targeting SAV1866 with potent inhibitors, it is possible to increase the intracellular concentration of antibiotics, thus restoring their efficacy against resistant bacteria, a process akin to using drug-resistant reversal agents [11,12,13,15,58,59]. On the other hand, MecA is a regulatory protein that plays a crucial role in regulating virulence in MRSA [7,8,9]. Targeting MecA provides an opportunity to develop anti-virulence therapies against MRSA, reducing its pathogenicity and enhancing the effectiveness of antibiotic treatments [60,61,62]. This could provide another chemotherapeutic approach to the treatment of MRSA infections [8]. Furthermore, compounds that strongly bind to MecA can inhibit its regulatory activities, disrupt the expression of virulence factors, and alleviate the severity of MRSA infections [7,8,9]. The identification of compounds with strong binding to both SAV1866 and MecA highlights the potential of natural compounds for developing novel therapeutic strategies [60,61].

One of the highly effective natural compounds identified in this study is 1′,2-Binaphthalen-4-one-2′,3-dimethyl-1,8′-epoxy-1,4′,5,5′,8,8′-hexahydroxy-8-O-β-glucopyranosyl-5′-O-β-xylopyranosyl(1→6)-β-glucopyranoside (SANC00524). This compound was obtained through the methanol extraction of *Diospyros lycioides* twigs, and has been shown to possess significant antimicrobial activity against *S. sanguis* and *Streptococcus mutans* [33]. Furthermore, we found that several other compounds, namely Cis-3,4-dihydrohamacanthin b (SANC00416), Bromodeoxytopsentin (SANC00415), (Bromotopsentin) SANC00413, and Spongotine A (SANC00414) [34] displayed strong affinity towards the MRSA SAV1866 protein. These Bis-indole alkaloids were extracted from *Topsentia pachastrelloides* and have been reported to possess antibacterial properties against MRSA. Additionally, these compounds have been observed to disrupt MRSA′s cell membranes and inhibit MRSA’s pyruvate kinase enzymatic activity [34]. In our study, we demonstrated the potent inhibitory effect of these compounds on the MRSA multidrug ABC transporter protein. Similarly, other compounds, including Mamegakinone (SANC00436), Diospyrin (SANC00434), Isodiospyrin (SANC00435), and Neodiospyrin (SANC00438), have exhibited strong affinity against the MRSA SAV1866 protein [35]. The work by Van der Kooy and colleagues demonstrated the inhibitory role of these compounds, derived from the roots of *Euclea natalensis*, against *Mycobacterium tuberculosis* [35]. Our current work provides evidence of their robust inhibition of the MRSA multidrug ABC transporter protein. The compounds (Martynoside) SANC01067 and Acteoside (SANC00370), extracted from the leaves of *Boscia albitrunca* [36], displayed remarkable affinity towards the MRSA SAV1866 protein. Additionally, Burttinone (SANC00940) and Abyssinone-V 4′-methyl ether (SANC00941), derived from the stem bark of *Erythrina caffra* Thunb [37], exhibited a strong affinity for the MRSA SAV1866 protein. These compounds, like the others mentioned above, possess potent activity against various Gram-negative and Gram-positive bacteria as well as fungi. We also identified an alkaloid compound, Nornantenine (SANC01101) [38], which strongly binds to MRSA SAV1866. This compound was extracted from the aerial parts of *Annona senegalensis* and has shown efficacy against *Streptococcus mutans* [38]. Lastly, Limonin (SANC01041), which we demonstrated to have a strong affinity against MRSA MecA but not SAV1866, was isolated from the root and stem bark of *V. glomerata* [39]. This compound has been shown to possess inhibitory activity against the growth of *Staphylococcus aureus* and *Shigella dysentrieae*. These findings highlight the diverse range of natural compounds with potent antimicrobial properties, specifically against MRSA and other bacterial pathogens.

While our findings provide valuable computational insights into the potential natural inhibitors targeting MRSA resistance proteins, the limitations must be acknowledged. Molecular docking offers meaningful predictions of binding modes and affinities, but it cannot capture the full biological complexity involved in drug discovery. Critical factors, such as metabolic stability, toxicity, bioavailability, off-target effects, and multi-target interactions, remain beyond the scope of docking and must be addressed in future work. Additionally, the predicted ligand–protein interactions, though promising, have not yet been validated experimentally. These results should therefore be interpreted as preliminary and hypothesis-generating, serving to prioritize compounds for follow-up in vitro and in vivo studies. We also recognize that the curated natural compound library used here, while useful, is limited in its chemical diversity, potentially constraining the range of candidate inhibitors identified. Expanding the compound library and incorporating complementary computational techniques, such as molecular dynamics (MDs) simulations, will be crucial for future studies to assess the temporal stability, conformational flexibility, and true therapeutic potential of these ligand–protein interactions.

## 5. Conclusions

This study demonstrates the utility of computational screening in the early stages of drug discovery, particularly for identifying natural compounds with potential antibacterial activity against MRSA. By employing molecular docking, we predicted the binding affinities and interaction profiles of selected compounds with key resistance-related proteins, notably SAV1866 and MecA. These targets are central to the antibiotic resistance mechanisms in MRSA, and the identification of strong binding interactions suggests promising avenues for further pharmacological development. While in silico methods cannot substitute for experimental validation, they serve as powerful tools for hypothesis generation and resource-efficient lead prioritization. Future work will focus on elucidating the specific mechanisms of action of these compounds, supported by molecular dynamics simulations and experimental assays. Additionally, preclinical and clinical studies will be essential to assess their safety, efficacy, and therapeutic potential. Overall, these findings provide a foundation for the development of novel antibacterial agents targeting multidrug-resistant *S. aureus*.

## Figures and Tables

**Table 1:** Summary of significant binding affinities of MRSA SAV1866 and MecA proteins with 15 natural compounds (from Supplementary Table S7).

SANDB ID	Source Organisms	MecA [Affinity (kcal/mol)]	SAV1866 [Affinity (kcal/mol)]
SANC00524	*Diospyros lycioides*	−8	−12.2
SANC00416	*Topsentia pachastrelloides*	−8.3	−11.3
SANC00436	*Euclea natalensis*	−8.4	−11.1
SANC00415	*Topsentia pachastrelloides*	−8.6	−10.7
SANC00434	*Euclea natalensis*	−8.7	−10.5
SANC00413	*Topsentia pachastrelloides*	−8.6	−10.4
SANC00435	*Euclea natalensis*	−8.2	−10.4
SANC00414	*Topsentia pachastrelloides*	−8.5	−10.4
SANC00438	*Euclea natalensis*	−8.2	−10.3
SANC01067	*Boscia albitrunca*	−8.4	−10.3
SANC00940	*Erythrina caffra*	−7.7	−10.3
SANC00941	*Erythrina caffra*	−7.7	−10.2
SANC00370	*Buddleja salviifolia*	−8.5	−10.2
SANC01101	*Annona senegalensis*	−7.3	−10.1
SANC01041	*Vepris glomerata*	−10	−9.7

## Data Availability

The original contributions presented in this study are included in the article/Supplementary Materials. Further inquiries can be directed to the corresponding author.

## Supplementary Materials

Supplementary tables and additional data supporting this study are available online at https://doi.org/10.6084/m9.figshare.29206415.v1 (accessed on 16 June 2025). Table S1. Binding affinity of MRSA MecA protein to 80 natural compounds. Table S2. Binding affinity of MRSA SAV1866 protein to 80 natural compounds. Table S3. Binding affinity of MRSA mgrA protein to 80 natural compounds. Table S4. Binding affinity of MRSA MepR protein to 80 natural compounds. Table S5. Binding affinity of MRSA arlR protein to 80 natural compounds. Table S6. Summary of binding affinity of MRSA antibiotic resistance proteins to 80 natural compounds. Table S7. Origin, classification, and significant binding affinity of MRSA SAV1866 and MecA proteins to 15 natural compounds. Table S8. Validation of selected high-affinity compounds binding to MecA and SAV1866 proteins.
